# Colorectal adenocarcinoma of the interposed colon after esophagectomy in infancy: a case report

**DOI:** 10.1093/jscr/rjae516

**Published:** 2024-08-23

**Authors:** Nina Schraps, Baris Mercanoglu, Anastasios Giannou, Thomas Witthöft, Thilo Hackert, Nathaniel Melling

**Affiliations:** Department of General, Visceral and Thoracic Surgery, University Medical Center Hamburg-Eppendorf, 20246 Hamburg, Germany; Department of General, Visceral and Thoracic Surgery, University Medical Center Hamburg-Eppendorf, 20246 Hamburg, Germany; Department of General, Visceral and Thoracic Surgery, University Medical Center Hamburg-Eppendorf, 20246 Hamburg, Germany; Gastroenterologische Gemeinschaftspraxis Stade, 21682 Stade, Germany; Department of General, Visceral and Thoracic Surgery, University Medical Center Hamburg-Eppendorf, 20246 Hamburg, Germany; Department of General, Visceral and Thoracic Surgery, University Medical Center Hamburg-Eppendorf, 20246 Hamburg, Germany

**Keywords:** colorectal adenocarcinoma, colon interposition, case report, gastric pull-up, esophagectomy

## Abstract

Colorectal carcinomas are a rare but possible complication in an interposed colonic segment used for reconstruction after esophagectomy. We report the case of a patient who underwent colonic interposition surgery in childhood due to esophageal atresia and was diagnosed with colorectal adenocarcinoma of the interposed colon ~57 years later. The patient underwent gastric pull-up after thoraco-abdominal resection of the colonic interposition en bloc with the adjacent remaining esophagus.

## Introduction

After esophagectomy due to benign or malignant diseases, colonic interposition can either be used as a primary method of reconstruction if the stomach is not suitable or available or as a secondary replacement after initial gastric pull-up [1]. However, the colon is also a popular organ for the formation of the neoesophagus in pediatric surgery [2, 3]. The indication for esophageal replacement in children includes long-gap esophageal atresia, strictures due to gastroesophageal reflux disease, as well as a number of rare diseases such as achalasia, epidermolysis bullosa, or candidiasis [2, 4]. Alternatives to colonic interposition include gastric pull-up or jejunal interposition [2, 4]. The reconstruction of the esophagus using colon is performed by maintaining its vascular pedicle. In addition to the anatomical advantages, such as the possible length of the reconstruction, it also offers the advantage of a low risk for reflux, good long-term satisfaction, and quality of life [3, 5]. Besides the more frequent complications such as anastomotic strictures or leakages, adenomas or adenocarcinomas can occur in rare cases [2, 6].

## Case report

**Figure 1:**
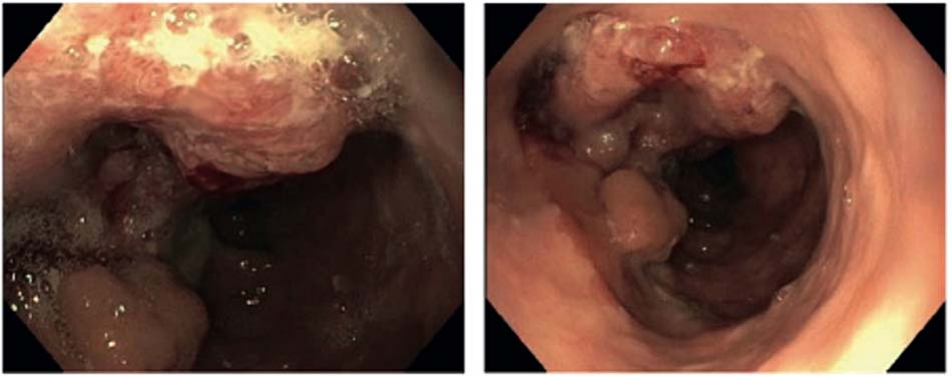
Endoscopic view showing a tumor of the transposed colon 23 cm from the incisors.

**Figure 2:**
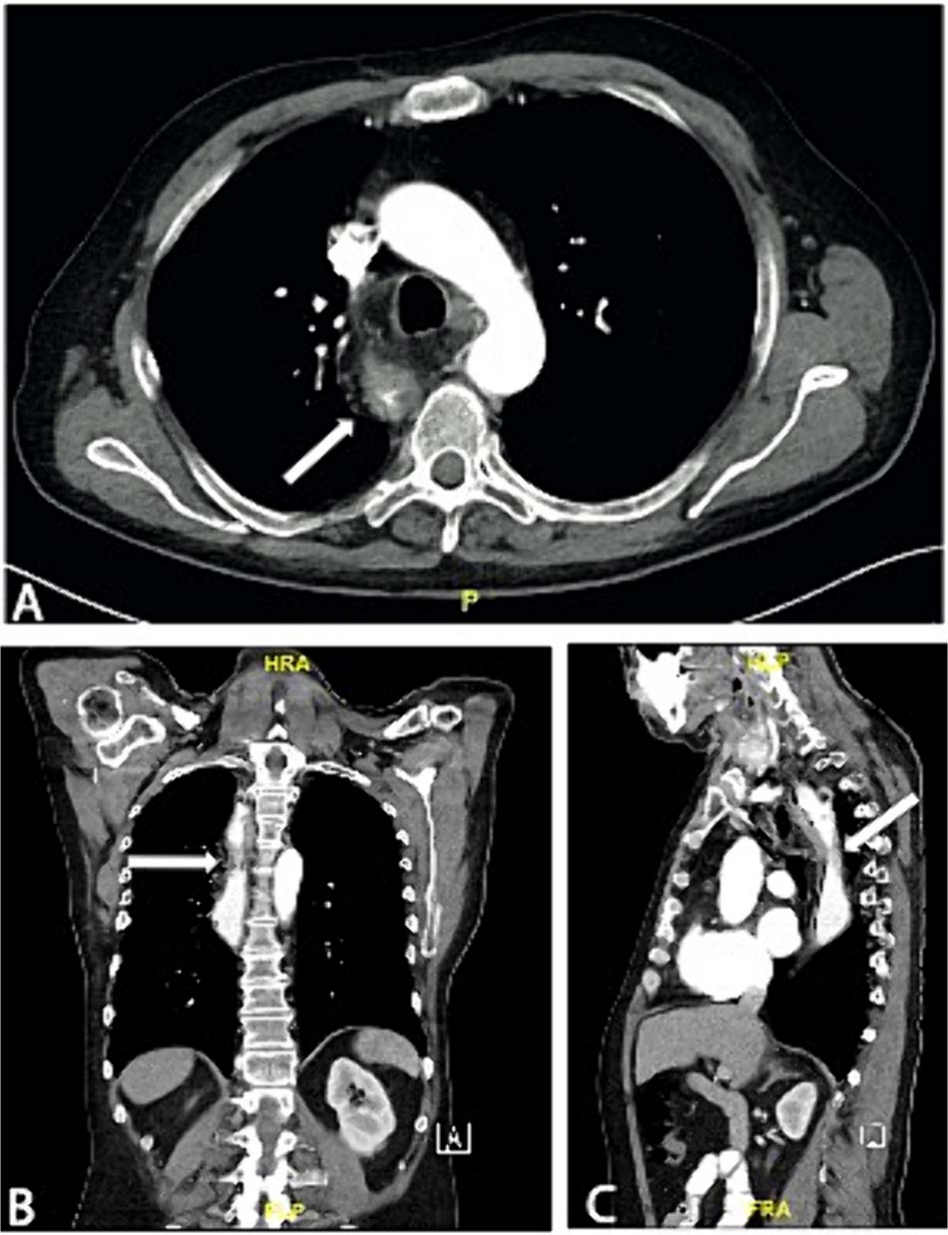
Axial (A), coronal (B), and sagittal slice (C) of a preoperative CT scan showing wall thickening of the neoesophagus proximal to the tracheal bifurcation.

We report the case of a 57-year-old patient who presented to our institution with histologically confirmed colorectal adenocarcinoma of the interposed colon after esophagectomy in infancy. At the age of 6 months, the patient underwent surgery for esophageal atresia with resection and colonic interposition. At the age of 4 years, a segment of the interposed colon was resected, most likely due to kinking. No records on these operations performed at a renowned institution in Germany around 1965 exist anymore. In December 2021, the patient presented with hematin reflux via an inserted gastric tube and underwent esophago-gastro-duodenoscopy, while he was hospitalized for myocardial infarction. A hemorrhage in the area of the intestinal interposition was clipped, but there were abnormalities of the mucosa of unclear dignity, which is why a upper endoscopy was repeated after discharge. The examination in August 2022 revealed a mass 23 cm from the incisors, occupying about one-third of the circumference (Fig. 1). The histological workup of the samples taken showed colonic mucosa including a colorectal adenocarcinoma. Staging CT scans of the thorax and abdomen revealed marked wall thickening of the neoesophagus proximal to the tracheal bifurcation over ~7.5 cm (Fig. 2). There was no evidence of suspicious pulmonary or hepatic lesions. Thus, the patient was planned for primary surgical resection, in accordance with the standard approach for colon cancer, after discussion in our multidisciplinary team meeting. The patient underwent thoracoabdominal esophagectomy with resection of the colonic interposition en bloc with adjacent healthy esophagus orally and distally. Reconstruction was performed by gastric pull-up. Due to the previous surgery, marked adhesions in the thorax were encountered and lead to an intraoperative injury of the lung, resulting in an atypical lung resection of the right upper lobe. Histological examination of the surgical specimen revealed moderately differentiated colorectal adenocarcinoma with lymphogenous metastasis (G2, pT3, pN2a (4/13), L0, V0, Pn0, R0, cM0). Approximately 2 months after surgery, adjuvant chemotherapy with CAPOX (capecitabine and oxaliplatin) was started.

Despite this, the first follow-up (at July 2023) revealed bipulmonary metastases. The treatment was switched to palliative systemic therapy with FOLFIRI (Folinic acid, Fluorouracil and Irinotecan). After starting systemic therapy and switching to third-line therapy, the patient continued to show pulmonary progression.

## Discussion

Colon interposition is a popular method for reconstruction after esophagectomy, both in adults with esophageal cancer, if the stomach is not available and also in children [1, 2]. Benign conditions that may require primary colonic interposition in childhood include long-gap esophageal atresia or strictures of the esophagus [2]. The transverse or left colon as an isoperistaltic conduit is used in preference to the right colon, partly due to the more reliable blood supply [4]. Frequent complications after colonic interposition are anastomotic leakages, gastroesophageal reflux, anastomotic strictures, and fistula formation [3]. Coopman *et al.* [7] investigated the long-term outcome of children after colon interposition; 53% showed complications within the first postoperative year, while long-term complications occurred in 84% of cases. Long-term complications included digestive problems, nutritional complications, or abnormal lung function [7]. A very rare complication, of which only a few cases have been described in the literature to date, is the formation of colorectal adenocarcinoma within the colonic interposition [6]. According to a systematic review, the development of mucosal changes occurs with a median of 8.5 years after the initial operation [6]. In our case, however, the cancer was detected 57 years after colonic interposition. While there are no general recommendations in the literature for the follow-up of a colonic interposition in children, the case presented here emphasizes the need for lifelong follow-up for early detection of possible pathological changes.

Colon cancer is one of the most common types of cancer worldwide in both women and men [8]. Therefore, apart from the regular colon cancer screening, which is performed from the age of 50 [9], colonoscopy is routinely performed before colon interposition to rule out pathologies, primarily colon cancers [10–12]. Besides preoperative screening, colon cancer as a late complication of colonic interposition indicates the need for further follow-up examinations in the form of upper GI endoscopies. These could for instance be carried out as part of regular colon cancer screening. Looking at the current guideline on screening for colorectal cancer, a repeat colonoscopy is recommended after 10 years if there are no pathological findings [9]. On the other hand, Heresbach *et al.* [13] showed a miss rate of 23.4% in the colonoscopic identification of neoplasia, which is why endoscopic control of the colonic interposition within 1 year after surgery for early detection and treatment has been suggested in literature [14]. According to a systematic review, the occurrence of interval pathologies is very rare, but the risk increases with a longer time interval to the initial operation [6]. The interval could be adjusted depending on individual risk factors, with more frequent follow-ups after 5 years. Risk factors that can be taken into consideration are a positive family history of colorectal cancer, colitis, or esophageal carcinoma as the indication for a colonic interposition, which has been shown to be associated with an increased risk of colon lesions [6, 15, 16].

In addition to genetic factors, environmental influences and dietary habits play a significant role in the development of colon cancer and are therefore considered further important risk factors [15]. The change in position of the colon leads to changes in the food composition to which the mucosa is exposed. In addition, there is the potential risk of gastric or bile acid reflux. There is evidence that bile acids in large quantities can have carcinogenic effect on the esophagus in Barrett’s esophagus and also on the colon [17].

The influence of environmental changes due to the new position of the colon could have a significant influence on mucosal changes and may be involved in the development of carcinoma.

In summary, our case demonstrates the need for a structured, life-long follow-up using upper endoscopy for early detection of pathological changes in the interposed colon.
